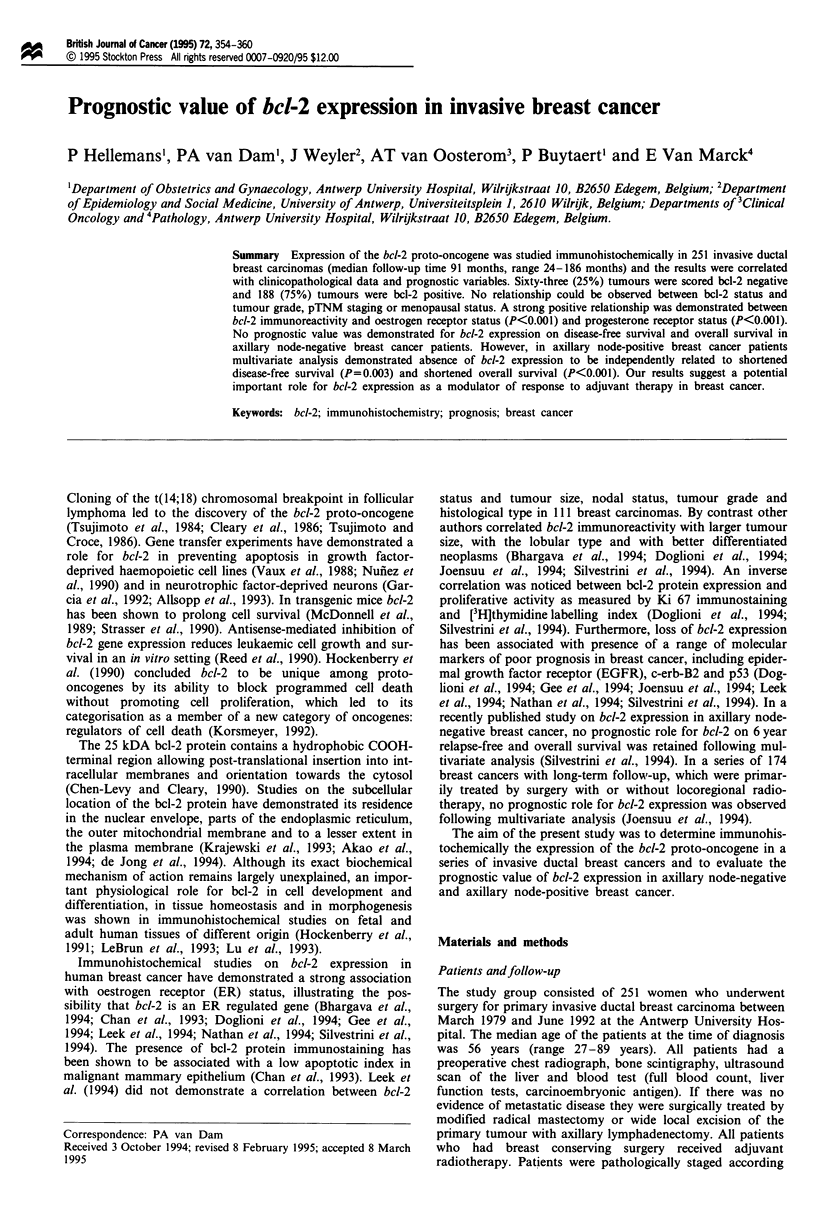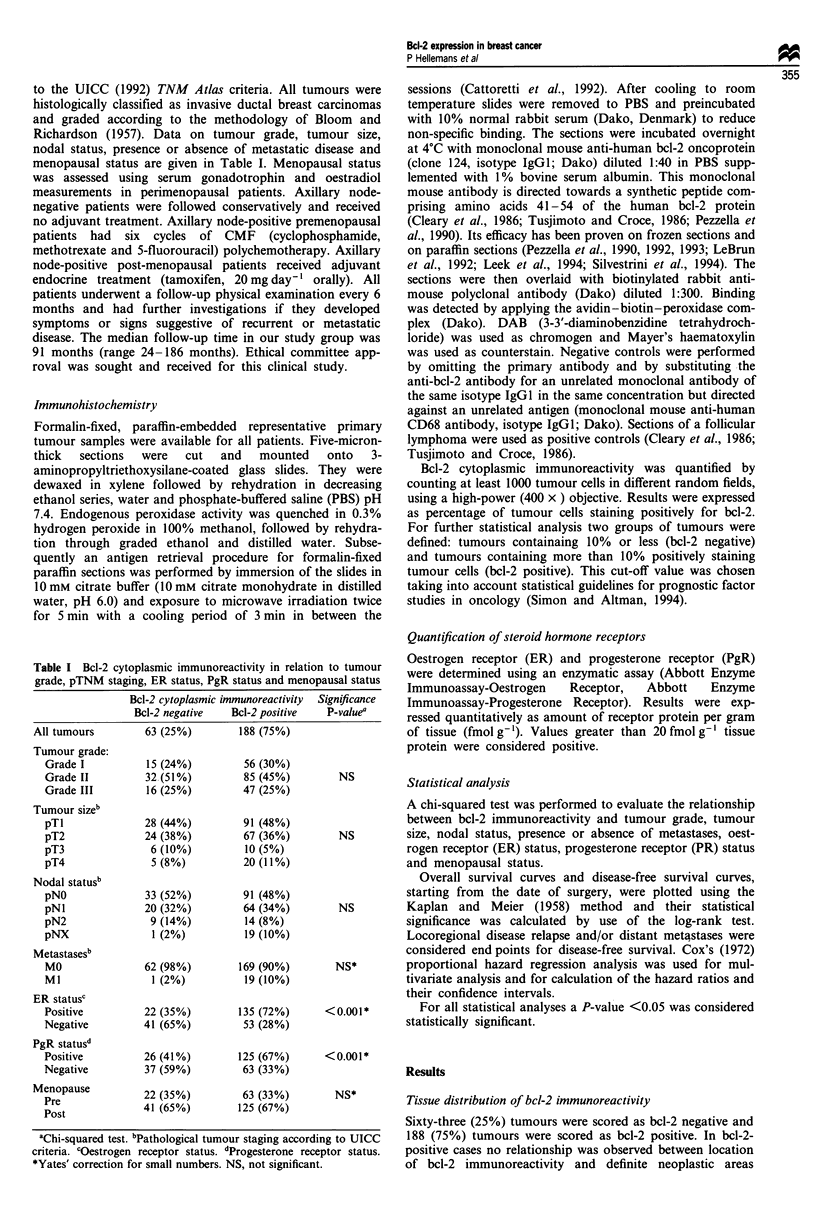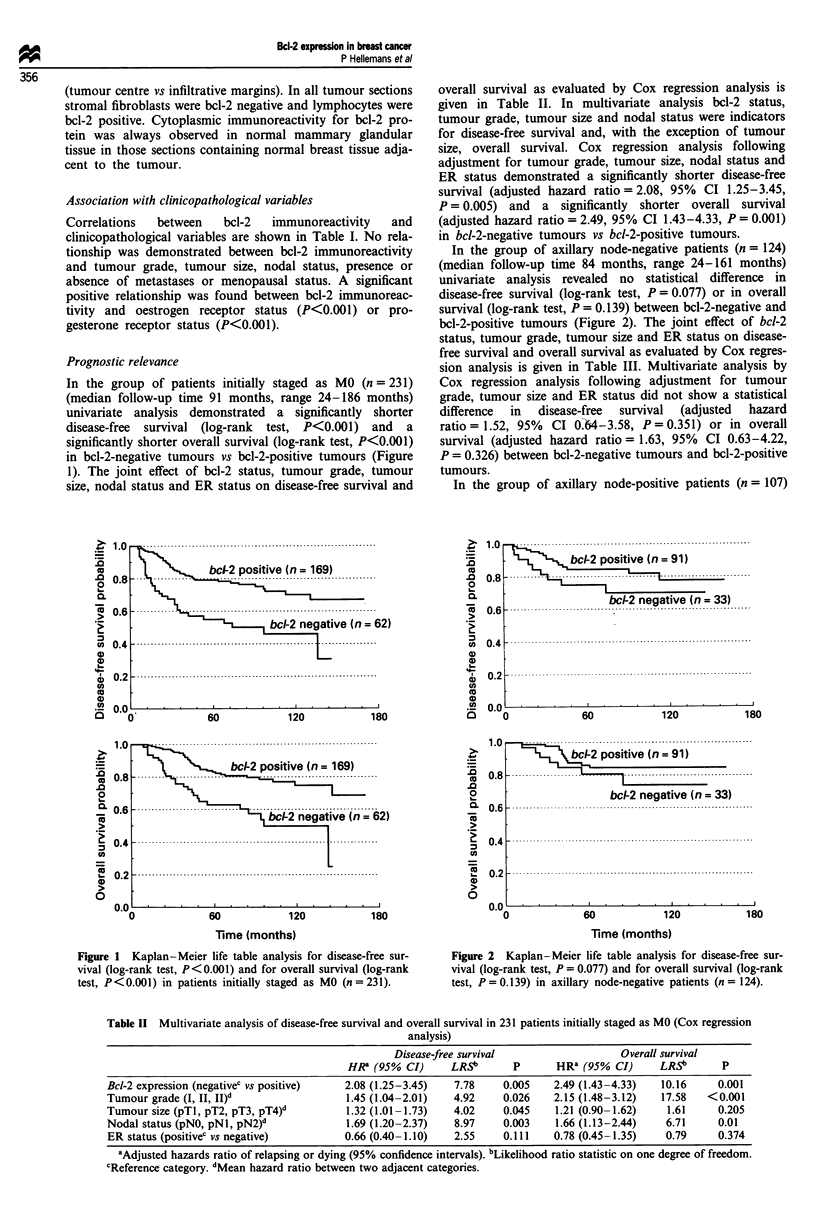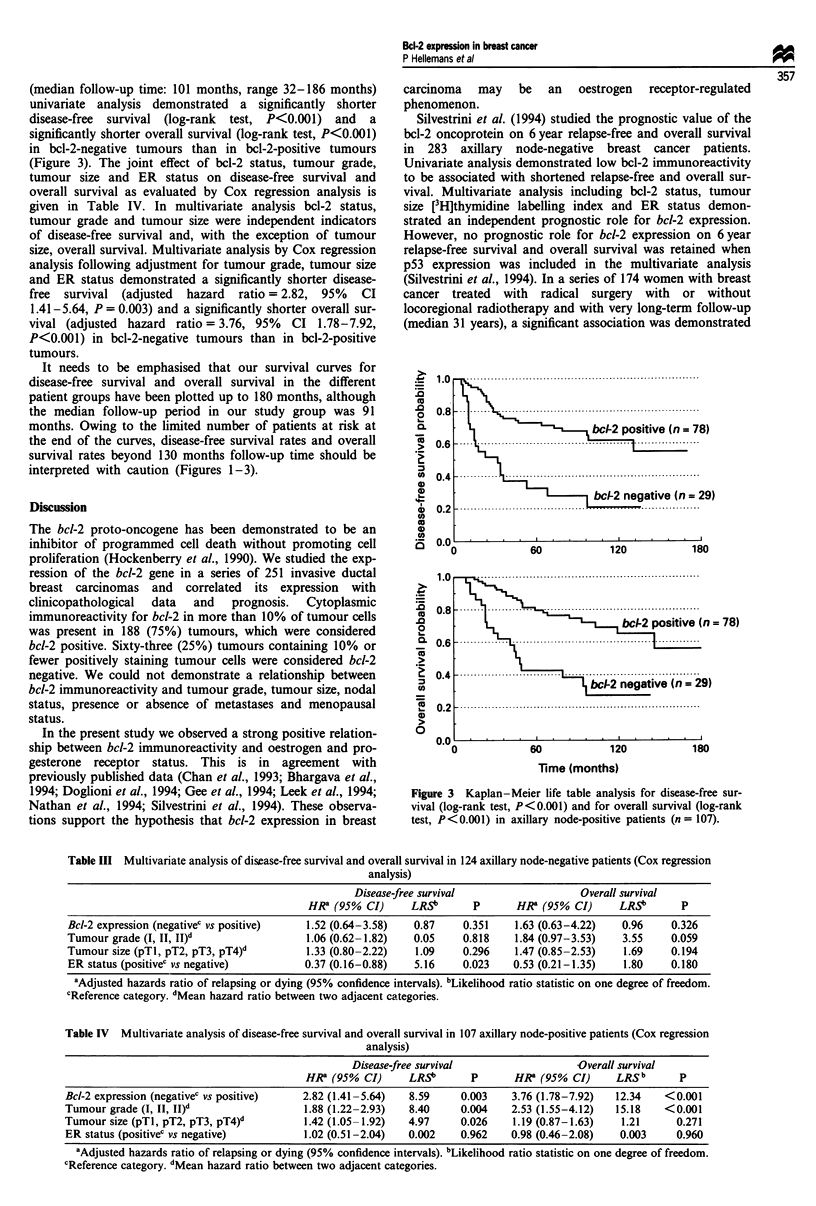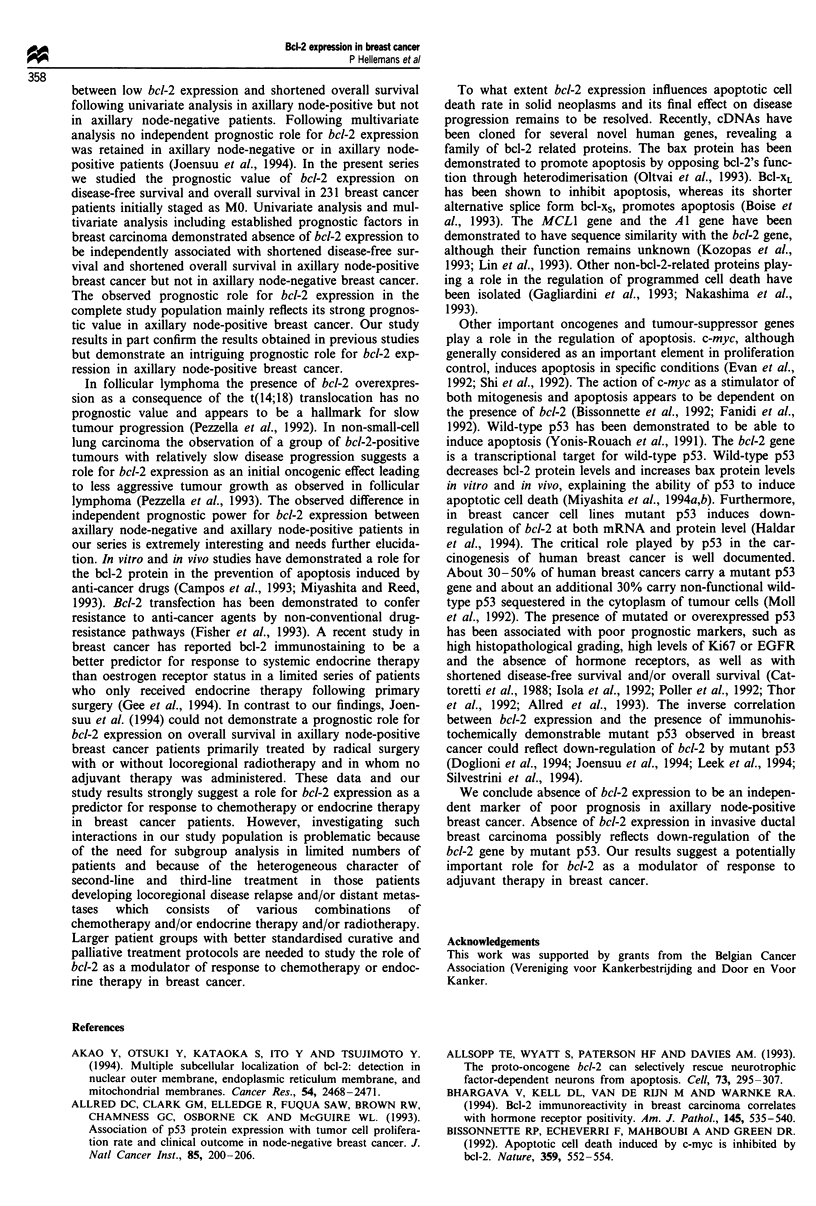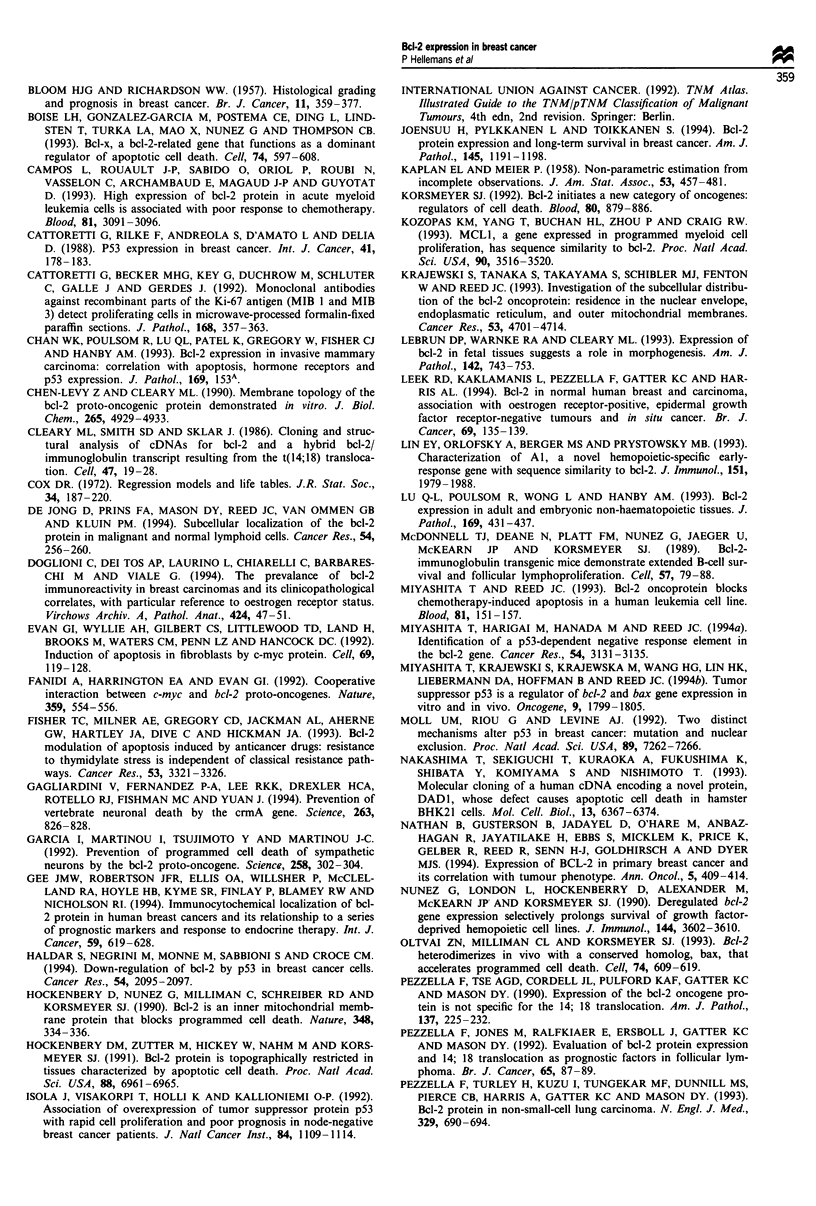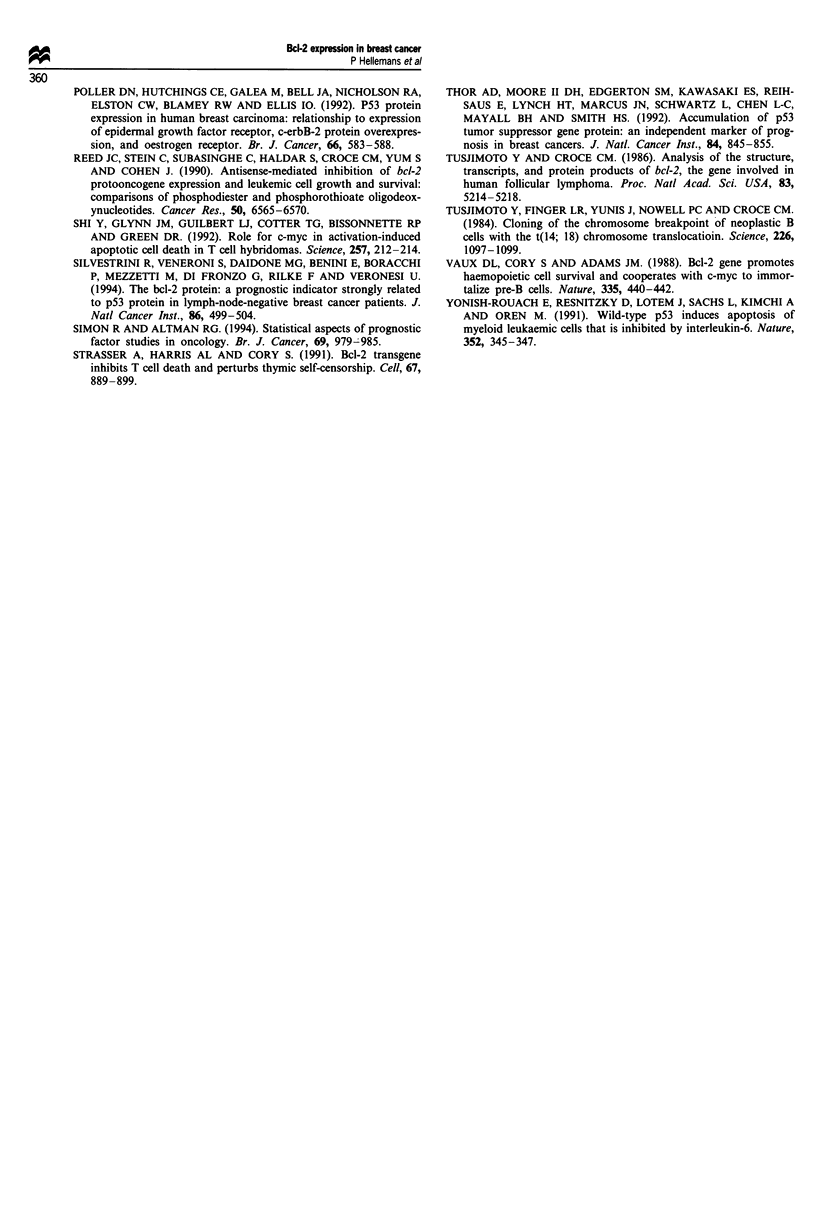# Prognostic value of bcl-2 expression in invasive breast cancer.

**DOI:** 10.1038/bjc.1995.338

**Published:** 1995-08

**Authors:** P. Hellemans, P. A. van Dam, J. Weyler, A. T. van Oosterom, P. Buytaert, E. Van Marck

**Affiliations:** Department of Obstetrics and Gynaecology, Antwerp University Hospital, Edegem, Belgium.

## Abstract

Expression of the bcl-2 proto-oncogene was studied immunohistochemically in 251 invasive ductal breast carcinomas (median follow-up time 91 months, range 24-186 months) and the results were correlated with clinicopathological data and prognostic variables. Sixty-three (25%) tumours were scored bcl-2 negative and 188 (75%) tumours were bcl-2 positive. No relationship could be observed between bcl-2 status and tumour grade, pTNM staging or menopausal status. A strong positive relationship was demonstrated between bcl-2 immunoreactivity and oestrogen receptor status (P < 0.001) and progesterone receptor status (P < 0.001). No prognostic value was demonstrated for bcl-2 expression on disease-free survival and overall survival in axillary node-negative breast cancer patients. However, in axillary node-positive breast cancer patients multivariate analysis demonstrated absence of bcl-2 expression to be independently related to shortened disease-free survival (P = 0.003) and shortened overall survival (P < 0.001). Our results suggest a potential important role for bcl-2 expression as a modulator of response to adjuvant therapy in breast cancer.


British Journal of Cancer (1995) 72, 354-360
© 1995 Stockton Press All rights reserved 0007-0920/95 $12.00

### Prognostic value of *bcl*-2 expression in invasive breast cancer

P Hellemans[1], PA van Dam[1], J Weyler[2], AT van Oosterom[3], P Buytaert[1] and E Van Marck[4]

[1]*Department of Obstetrics and Gynaecology, Antwerp University Hospital, Wilrijkstraat 10, B2650 Edegem, Belgium;* [2]*Department of Epidemiology and Social Medicine, University of Antwerp, Universiteitsplein 1, 2610 Wilrijk, Belgium; Departments of* [3]*Clinical Oncology and* [4]*Pathology, Antwerp University Hospital, Wilrijkstraat 10, B2650 Edegem, Belgium.*

**Summary** Expression of the *bcl*-2 proto-oncogene was studied immunohistochemically in 251 invasive ductal breast carcinomas (median follow-up time 91 months, range 24-186 months) and the results were correlated with clinicopathological data and prognostic variables. Sixty-three (25%) tumours were scored bcl-2 negative and 188 (75%) tumours were bcl-2 positive. No relationship could be observed between bcl-2 status and tumour grade, pTNM staging or menopausal status. A strong positive relationship was demonstrated between *bcl*-2 immunoreactivity and oestrogen receptor status ($P<0.001$) and progesterone receptor status ($P<0.001$). No prognostic value was demonstrated for *bcl*-2 expression on disease-free survival and overall survival in axillary node-negative breast cancer patients. However, in axillary node-positive breast cancer patients multivariate analysis demonstrated absence of *bcl*-2 expression to be independently related to shortened disease-free survival ($P=0.003$) and shortened overall survival ($P<0.001$). Our results suggest a potential important role for *bcl*-2 expression as a modulator of response to adjuvant therapy in breast cancer.

**Keywords:** *bcl*-2; immunohistochemistry; prognosis; breast cancer

Cloning of the t(14;18) chromosomal breakpoint in follicular lymphoma led to the discovery of the *bcl*-2 proto-oncogene (Tsujimoto *et al.*, 1984; Cleary *et al.*, 1986; Tsujimoto and Croce, 1986). Gene transfer experiments have demonstrated a role for *bcl*-2 in preventing apoptosis in growth factor-deprived haemopoietic cell lines (Vaux *et al.*, 1988; Nuñez *et al.*, 1990) and in neurotrophic factor-deprived neurons (Garcia *et al.*, 1992; Allsopp *et al.*, 1993). In transgenic mice *bcl*-2 has been shown to prolong cell survival (McDonnell *et al.*, 1989; Strasser *et al.*, 1990). Antisense-mediated inhibition of *bcl*-2 gene expression reduces leukaemic cell growth and survival in an *in vitro* setting (Reed *et al.*, 1990). Hockenberry *et al.* (1990) concluded *bcl*-2 to be unique among proto-oncogenes by its ability to block programmed cell death without promoting cell proliferation, which led to its categorisation as a member of a new category of oncogenes: regulators of cell death (Korsmeyer, 1992).

The 25 kDA bcl-2 protein contains a hydrophobic COOH-terminal region allowing post-translational insertion into intracellular membranes and orientation towards the cytosol (Chen-Levy and Cleary, 1990). Studies on the subcellular location of the bcl-2 protein have demonstrated its residence in the nuclear envelope, parts of the endoplasmic reticulum, the outer mitochondrial membrane and to a lesser extent in the plasma membrane (Krajewski *et al.*, 1993; Akao *et al.*, 1994; de Jong *et al.*, 1994). Although its exact biochemical mechanism of action remains largely unexplained, an important physiological role for bcl-2 in cell development and differentiation, in tissue homeostasis and in morphogenesis was shown in immunohistochemical studies on fetal and adult human tissues of different origin (Hockenberry *et al.*, 1991; LeBrun *et al.*, 1993; Lu *et al.*, 1993).

Immunohistochemical studies on *bcl*-2 expression in human breast cancer have demonstrated a strong association with oestrogen receptor (ER) status, illustrating the possibility that *bcl*-2 is an ER regulated gene (Bhargava *et al.*, 1994; Chan *et al.*, 1993; Doglioni *et al.*, 1994; Gee *et al.*, 1994; Leek *et al.*, 1994; Nathan *et al.*, 1994; Silvestrini *et al.*, 1994). The presence of bcl-2 protein immunostaining has been shown to be associated with a low apoptotic index in malignant mammary epithelium (Chan *et al.*, 1993). Leek *et al.* (1994) did not demonstrate a correlation between *bcl*-2 status and tumour size, nodal status, tumour grade and histological type in 111 breast carcinomas. By contrast other authors correlated *bcl*-2 immunoreactivity with larger tumour size, with the lobular type and with better differentiated neoplasms (Bhargava *et al.*, 1994; Doglioni *et al.*, 1994; Joensuu *et al.*, 1994; Silvestrini *et al.*, 1994). An inverse correlation was noticed between bcl-2 protein expression and proliferative activity as measured by Ki 67 immunostaining and [$^3$H]thymidine labelling index (Doglioni *et al.*, 1994; Silvestrini *et al.*, 1994). Furthermore, loss of *bcl*-2 expression has been associated with presence of a range of molecular markers of poor prognosis in breast cancer, including epidermal growth factor receptor (EGFR), c-erb-B2 and p53 (Doglioni *et al.*, 1994; Gee *et al.*, 1994; Joensuu *et al.*, 1994; Leek *et al.*, 1994; Nathan *et al.*, 1994; Silvestrini *et al.*, 1994). In a recently published study on *bcl*-2 expression in axillary node-negative breast cancer, no prognostic role for *bcl*-2 on 6 year relapse-free and overall survival was retained following multivariate analysis (Silvestrini *et al.*, 1994). In a series of 174 breast cancers with long-term follow-up, which were primarily treated by surgery with or without locoregional radiotherapy, no prognostic role for *bcl*-2 expression was observed following multivariate analysis (Joensuu *et al.*, 1994).

The aim of the present study was to determine immunohistochemically the expression of the *bcl*-2 proto-oncogene in a series of invasive ductal breast cancers and to evaluate the prognostic value of *bcl*-2 expression in axillary node-negative and axillary node-positive breast cancer.

### Materials and methods

### *Patients and follow-up*

The study group consisted of 251 women who underwent surgery for primary invasive ductal breast carcinoma between March 1979 and June 1992 at the Antwerp University Hospital. The median age of the patients at the time of diagnosis was 56 years (range 27-89 years). All patients had a preoperative chest radiograph, bone scintigraphy, ultrasound scan of the liver and blood test (full blood count, liver function tests, carcinoembryonic antigen). If there was no evidence of metastatic disease they were surgically treated by modified radical mastectomy or wide local excision of the primary tumour with axillary lymphadenectomy. All patients who had breast conserving surgery received adjuvant radiotherapy. Patients were pathologically staged according

Correspondence: PA van Dam
Received 3 October 1994; revised 8 February 1995; accepted 8 March 1995

to the UICC (1992) *TNM Atlas* criteria. All tumours were histologically classified as invasive ductal breast carcinomas and graded according to the methodology of Bloom and Richardson (1957). Data on tumour grade, tumour size, nodal status, presence or absence of metastatic disease and menopausal status are given in Table I. Menopausal status was assessed using serum gonadotrophin and oestradiol measurements in perimenopausal patients. Axillary node-negative patients were followed conservatively and received no adjuvant treatment. Axillary node-positive premenopausal patients had six cycles of CMF (cyclophosphamide, methotrexate and 5-fluorouracil) polychemotherapy. Axillary node-positive post-menopausal patients received adjuvant endocrine treatment (tamoxifen, 20 mg $day^{-1}$ orally). All patients underwent a follow-up physical examination every 6 months and had further investigations if they developed symptoms or signs suggestive of recurrent or metastatic disease. The median follow-up time in our study group was 91 months (range 24–186 months). Ethical committee approval was sought and received for this clinical study.

### *Immunohistochemistry*

Formalin-fixed, paraffin-embedded representative primary tumour samples were available for all patients. Five-micron-thick sections were cut and mounted onto 3-aminopropyltriethoxysilane-coated glass slides. They were dewaxed in xylene followed by rehydration in decreasing ethanol series, water and phosphate-buffered saline (PBS) pH 7.4. Endogenous peroxidase activity was quenched in 0.3% hydrogen peroxide in 100% methanol, followed by rehydration through graded ethanol and distilled water. Subsequently an antigen retrieval procedure for formalin-fixed paraffin sections was performed by immersion of the slides in 10 mM citrate buffer (10 mM citrate monohydrate in distilled water, pH 6.0) and exposure to microwave irradiation twice for 5 min with a cooling period of 3 min in between the sessions (Cattoretti *et al.*, 1992). After cooling to room temperature slides were removed to PBS and preincubated with 10% normal rabbit serum (Dako, Denmark) to reduce non-specific binding. The sections were incubated overnight at 4°C with monoclonal mouse anti-human bcl-2 oncoprotein (clone 124, isotype IgG1; Dako) diluted 1:40 in PBS supplemented with 1% bovine serum albumin. This monoclonal mouse antibody is directed towards a synthetic peptide comprising amino acids 41–54 of the human bcl-2 protein (Cleary *et al.*, 1986; Tusjimoto and Croce, 1986; Pezzella *et al.*, 1990). Its efficacy has been proven on frozen sections and on paraffin sections (Pezzella *et al.*, 1990, 1992, 1993; LeBrun *et al.*, 1992; Leek *et al.*, 1994; Silvestrini *et al.*, 1994). The sections were then overlaid with biotinylated rabbit anti-mouse polyclonal antibody (Dako) diluted 1:300. Binding was detected by applying the avidin–biotin–peroxidase complex (Dako). DAB (3-3′-diaminobenzidine tetrahydrochloride) was used as chromogen and Mayer's haematoxylin was used as counterstain. Negative controls were performed by omitting the primary antibody and by substituting the anti-bcl-2 antibody for an unrelated monoclonal antibody of the same isotype IgG1 in the same concentration but directed against an unrelated antigen (monoclonal mouse anti-human CD68 antibody, isotype IgG1; Dako). Sections of a follicular lymphoma were used as positive controls (Cleary *et al.*, 1986; Tusjimoto and Croce, 1986).

Bcl-2 cytoplasmic immunoreactivity was quantified by counting at least 1000 tumour cells in different random fields, using a high-power (400 × ) objective. Results were expressed as percentage of tumour cells staining positively for bcl-2. For further statistical analysis two groups of tumours were defined: tumours containaing 10% or less (bcl-2 negative) and tumours containing more than 10% positively staining tumour cells (bcl-2 positive). This cut-off value was chosen taking into account statistical guidelines for prognostic factor studies in oncology (Simon and Altman, 1994).

**Table I** Bcl-2 cytoplasmic immunoreactivity in relation to tumour grade, pTNM staging, ER status, PgR status and menopausal status

| | *Bcl-2 cytoplasmic immunoreactivity* Bcl-*2 negative* | Bcl-*2 positive* | *Significance* P-*value*[a] |
|---|---|---|---|
| All tumours | 63 (25%) | 188 (75%) | |
| Tumour grade: | | | |
| Grade I | 15 (24%) | 56 (30%) | |
| Grade II | 32 (51%) | 85 (45%) | NS |
| Grade III | 16 (25%) | 47 (25%) | |
| Tumour size[b] | | | |
| pT1 | 28 (44%) | 91 (48%) | |
| pT2 | 24 (38%) | 67 (36%) | NS |
| pT3 | 6 (10%) | 10 (5%) | |
| pT4 | 5 (8%) | 20 (11%) | |
| Nodal status[b] | | | |
| pN0 | 33 (52%) | 91 (48%) | |
| pN1 | 20 (32%) | 64 (34%) | NS |
| pN2 | 9 (14%) | 14 (8%) | |
| pNX | 1 (2%) | 19 (10%) | |
| Metastases[b] | | | |
| M0 | 62 (98%) | 169 (90%) | NS* |
| M1 | 1 (2%) | 19 (10%) | |
| ER status[c] | | | |
| Positive | 22 (35%) | 135 (72%) | <0.001* |
| Negative | 41 (65%) | 53 (28%) | |
| PgR status[d] | | | |
| Positive | 26 (41%) | 125 (67%) | <0.001* |
| Negative | 37 (59%) | 63 (33%) | |
| Menopause | | | |
| Pre | 22 (35%) | 63 (33%) | NS* |
| Post | 41 (65%) | 125 (67%) | |

[a]Chi-squared test. [b]Pathological tumour staging according to UICC criteria. [c]Oestrogen receptor status. [d]Progesterone receptor status. *Yates' correction for small numbers. NS, not significant.

### *Quantification of steroid hormone receptors*

Oestrogen receptor (ER) and progesterone receptor (PgR) were determined using an enzymatic assay (Abbott Enzyme Immunoassay-Oestrogen Receptor, Abbott Enzyme Immunoassay-Progesterone Receptor). Results were expressed quantitatively as amount of receptor protein per gram of tissue (fmol $g^{-1}$). Values greater than 20 fmol $g^{-1}$ tissue protein were considered positive.

### *Statistical analysis*

A chi-squared test was performed to evaluate the relationship between bcl-2 immunoreactivity and tumour grade, tumour size, nodal status, presence or absence of metastases, oestrogen receptor (ER) status, progesterone receptor (PR) status and menopausal status.

Overall survival curves and disease-free survival curves, starting from the date of surgery, were plotted using the Kaplan and Meier (1958) method and their statistical significance was calculated by use of the log-rank test. Locoregional disease relapse and/or distant metastases were considered end points for disease-free survival. Cox's (1972) proportional hazard regression analysis was used for multivariate analysis and for calculation of the hazard ratios and their confidence intervals.

For all statistical analyses a *P*-value <0.05 was considered statistically significant.

### Results

### *Tissue distribution of bcl-2 immunoreactivity*

Sixty-three (25%) tumours were scored as bcl-2 negative and 188 (75%) tumours were scored as bcl-2 positive. In bcl-2-positive cases no relationship was observed between location of bcl-2 immunoreactivity and definite neoplastic areas

(tumour centre *vs* infiltrative margins). In all tumour sections stromal fibroblasts were bcl-2 negative and lymphocytes were bcl-2 positive. Cytoplasmic immunoreactivity for bcl-2 protein was always observed in normal mammary glandular tissue in those sections containing normal breast tissue adjacent to the tumour.

### *Association with clinicopathological variables*

Correlations between bcl-2 immunoreactivity and clinicopathological variables are shown in Table I. No relationship was demonstrated between bcl-2 immunoreactivity and tumour grade, tumour size, nodal status, presence or absence of metastases or menopausal status. A significant positive relationship was found between bcl-2 immunoreactivity and oestrogen receptor status ($P<0.001$) or progesterone receptor status ($P<0.001$).

### *Prognostic relevance*

In the group of patients initially staged as M0 ($n=231$) (median follow-up time 91 months, range 24–186 months) univariate analysis demonstrated a significantly shorter disease-free survival (log-rank test, $P<0.001$) and a significantly shorter overall survival (log-rank test, $P<0.001$) in bcl-2-negative tumours *vs* bcl-2-positive tumours (Figure 1). The joint effect of bcl-2 status, tumour grade, tumour size, nodal status and ER status on disease-free survival and overall survival as evaluated by Cox regression analysis is given in Table II. In multivariate analysis bcl-2 status, tumour grade, tumour size and nodal status were indicators for disease-free survival and, with the exception of tumour size, overall survival. Cox regression analysis following adjustment for tumour grade, tumour size, nodal status and ER status demonstrated a significantly shorter disease-free survival (adjusted hazard ratio = 2.08, 95% CI 1.25–3.45, $P=0.005$) and a significantly shorter overall survival (adjusted hazard ratio = 2.49, 95% CI 1.43–4.33, $P=0.001$) in *bcl*-2-negative tumours *vs* *bcl*-2-positive tumours.

In the group of axillary node-negative patients ($n=124$) (median follow-up time 84 months, range 24–161 months) univariate analysis revealed no statistical difference in disease-free survival (log-rank test, $P=0.077$) or in overall survival (log-rank test, $P=0.139$) between bcl-2-negative and bcl-2-positive tumours (Figure 2). The joint effect of *bcl*-2 status, tumour grade, tumour size and ER status on disease-free survival and overall survival as evaluated by Cox regression analysis is given in Table III. Multivariate analysis by Cox regression analysis following adjustment for tumour grade, tumour size and ER status did not show a statistical difference in disease-free survival (adjusted hazard ratio = 1.52, 95% CI 0.64–3.58, $P=0.351$) or in overall survival (adjusted hazard ratio = 1.63, 95% CI 0.63–4.22, $P=0.326$) between bcl-2-negative tumours and bcl-2-positive tumours.

In the group of axillary node-positive patients ($n=107$)

**Figure 1** Kaplan–Meier life table analysis for disease-free survival (log-rank test, $P<0.001$) and for overall survival (log-rank test, $P<0.001$) in patients initially staged as M0 ($n=231$).

**Figure 2** Kaplan–Meier life table analysis for disease-free survival (log-rank test, $P=0.077$) and for overall survival (log-rank test, $P=0.139$) in axillary node-negative patients ($n=124$).

**Table II** Multivariate analysis of disease-free survival and overall survival in 231 patients initially staged as M0 (Cox regression analysis)

| | *Disease-free survival* | | | *Overall survival* | | |
|---|---|---|---|---|---|---|
| | *HR*[a] *(95% CI)* | *LRS*[b] | P | HR[a] *(95% CI)* | *LRS*[b] | P |
| *Bcl*-2 expression (negative[c] *vs* positive) | 2.08 (1.25–3.45) | 7.78 | 0.005 | 2.49 (1.43–4.33) | 10.16 | 0.001 |
| Tumour grade (I, II, II)[d] | 1.45 (1.04–2.01) | 4.92 | 0.026 | 2.15 (1.48–3.12) | 17.58 | <0.001 |
| Tumour size (pT1, pT2, pT3, pT4)[d] | 1.32 (1.01–1.73) | 4.02 | 0.045 | 1.21 (0.90–1.62) | 1.61 | 0.205 |
| Nodal status (pN0, pN1, pN2)[d] | 1.69 (1.20–2.37) | 8.97 | 0.003 | 1.66 (1.13–2.44) | 6.71 | 0.01 |
| ER status (positive[c] *vs* negative) | 0.66 (0.40–1.10) | 2.55 | 0.111 | 0.78 (0.45–1.35) | 0.79 | 0.374 |

[a]Adjusted hazards ratio of relapsing or dying (95% confidence intervals). [b]Likelihood ratio statistic on one degree of freedom. [c]Reference category. [d]Mean hazard ratio between two adjacent categories.

(median follow-up time: 101 months, range 32–186 months) univariate analysis demonstrated a significantly shorter disease-free survival (log-rank test, $P<0.001$) and a significantly shorter overall survival (log-rank test, $P<0.001$) in bcl-2-negative tumours than in bcl-2-positive tumours (Figure 3). The joint effect of bcl-2 status, tumour grade, tumour size and ER status on disease-free survival and overall survival as evaluated by Cox regression analysis is given in Table IV. In multivariate analysis bcl-2 status, tumour grade and tumour size were independent indicators of disease-free survival and, with the exception of tumour size, overall survival. Multivariate analysis by Cox regression analysis following adjustment for tumour grade, tumour size and ER status demonstrated a significantly shorter disease-free survival (adjusted hazard ratio = 2.82, 95% CI 1.41–5.64, $P=0.003$) and a significantly shorter overall survival (adjusted hazard ratio = 3.76, 95% CI 1.78–7.92, $P<0.001$) in bcl-2-negative tumours than in bcl-2-positive tumours.

It needs to be emphasised that our survival curves for disease-free survival and overall survival in the different patient groups have been plotted up to 180 months, although the median follow-up period in our study group was 91 months. Owing to the limited number of patients at risk at the end of the curves, disease-free survival rates and overall survival rates beyond 130 months follow-up time should be interpreted with caution (Figures 1–3).

### Discussion

The *bcl*-2 proto-oncogene has been demonstrated to be an inhibitor of programmed cell death without promoting cell proliferation (Hockenberry *et al.*, 1990). We studied the expression of the *bcl*-2 gene in a series of 251 invasive ductal breast carcinomas and correlated its expression with clinicopathological data and prognosis. Cytoplasmic immunoreactivity for *bcl*-2 in more than 10% of tumour cells was present in 188 (75%) tumours, which were considered *bcl*-2 positive. Sixty-three (25%) tumours containing 10% or fewer positively staining tumour cells were considered *bcl*-2 negative. We could not demonstrate a relationship between *bcl*-2 immunoreactivity and tumour grade, tumour size, nodal status, presence or absence of metastases and menopausal status.

In the present study we observed a strong positive relationship between *bcl*-2 immunoreactivity and oestrogen and progesterone receptor status. This is in agreement with previously published data (Chan *et al.*, 1993; Bhargava *et al.*, 1994; Doglioni *et al.*, 1994; Gee *et al.*, 1994; Leek *et al.*, 1994; Nathan *et al.*, 1994; Silvestrini *et al.*, 1994). These observations support the hypothesis that *bcl*-2 expression in breast carcinoma may be an oestrogen receptor-regulated phenomenon.

Silvestrini *et al.* (1994) studied the prognostic value of the bcl-2 oncoprotein on 6 year relapse-free and overall survival in 283 axillary node-negative breast cancer patients. Univariate analysis demonstrated low bcl-2 immunoreactivity to be associated with shortened relapse-free and overall survival. Multivariate analysis including bcl-2 status, tumour size [$^3$H]thymidine labelling index and ER status demonstrated an independent prognostic role for *bcl*-2 expression. However, no prognostic role for *bcl*-2 expression on 6 year relapse-free survival and overall survival was retained when p53 expression was included in the multivariate analysis (Silvestrini *et al.*, 1994). In a series of 174 women with breast cancer treated with radical surgery with or without locoregional radiotherapy and with very long-term follow-up (median 31 years), a significant association was demonstrated

**Figure 3** Kaplan–Meier life table analysis for disease-free survival (log-rank test, $P<0.001$) and for overall survival (log-rank test, $P<0.001$) in axillary node-positive patients ($n=107$).

**Table III** Multivariate analysis of disease-free survival and overall survival in 124 axillary node-negative patients (Cox regression analysis)

| | *Disease-free survival* | | | *Overall survival* | | |
|---|---|---|---|---|---|---|
| | *HR*[a] *(95% CI)* | *LRS*[b] | P | *HR*[a] *(95% CI)* | *LRS*[b] | P |
| *Bcl*-2 expression (negative[c] *vs* positive) | 1.52 (0.64–3.58) | 0.87 | 0.351 | 1.63 (0.63–4.22) | 0.96 | 0.326 |
| Tumour grade (I, II, II)[d] | 1.06 (0.62–1.82) | 0.05 | 0.818 | 1.84 (0.97–3.53) | 3.55 | 0.059 |
| Tumour size (pT1, pT2, pT3, pT4)[d] | 1.33 (0.80–2.22) | 1.09 | 0.296 | 1.47 (0.85–2.53) | 1.69 | 0.194 |
| ER status (positive[c] *vs* negative) | 0.37 (0.16–0.88) | 5.16 | 0.023 | 0.53 (0.21–1.35) | 1.80 | 0.180 |

[a]Adjusted hazards ratio of relapsing or dying (95% confidence intervals). [b]Likelihood ratio statistic on one degree of freedom. [c]Reference category. [d]Mean hazard ratio between two adjacent categories.

**Table IV** Multivariate analysis of disease-free survival and overall survival in 107 axillary node-positive patients (Cox regression analysis)

| | *Disease-free survival* | | | *Overall survival* | | |
|---|---|---|---|---|---|---|
| | *HR*[a] *(95% CI)* | *LRS*[b] | P | *HR*[a] *(95% CI)* | *LRS*[b] | P |
| *Bcl*-2 expression (negative[c] *vs* positive) | 2.82 (1.41–5.64) | 8.59 | 0.003 | 3.76 (1.78–7.92) | 12.34 | <0.001 |
| Tumour grade (I, II, II)[d] | 1.88 (1.22–2.93) | 8.40 | 0.004 | 2.53 (1.55–4.12) | 15.18 | <0.001 |
| Tumour size (pT1, pT2, pT3, pT4)[d] | 1.42 (1.05–1.92) | 4.97 | 0.026 | 1.19 (0.87–1.63) | 1.21 | 0.271 |
| ER status (positive[c] *vs* negative) | 1.02 (0.51–2.04) | 0.002 | 0.962 | 0.98 (0.46–2.08) | 0.003 | 0.960 |

[a]Adjusted hazards ratio of relapsing or dying (95% confidence intervals). [b]Likelihood ratio statistic on one degree of freedom. [c]Reference category. [d]Mean hazard ratio between two adjacent categories.

between low *bcl*-2 expression and shortened overall survival following univariate analysis in axillary node-positive but not in axillary node-negative patients. Following multivariate analysis no independent prognostic role for *bcl*-2 expression was retained in axillary node-negative or in axillary node-positive patients (Joensuu *et al.*, 1994). In the present series we studied the prognostic value of *bcl*-2 expression on disease-free survival and overall survival in 231 breast cancer patients initially staged as M0. Univariate analysis and multivariate analysis including established prognostic factors in breast carcinoma demonstrated absence of *bcl*-2 expression to be independently associated with shortened disease-free survival and shortened overall survival in axillary node-positive breast cancer but not in axillary node-negative breast cancer. The observed prognostic role for *bcl*-2 expression in the complete study population mainly reflects its strong prognostic value in axillary node-positive breast cancer. Our study results in part confirm the results obtained in previous studies but demonstrate an intriguing prognostic role for *bcl*-2 expression in axillary node-positive breast cancer.

In follicular lymphoma the presence of *bcl*-2 overexpression as a consequence of the t(14;18) translocation has no prognostic value and appears to be a hallmark for slow tumour progression (Pezzella *et al.*, 1992). In non-small-cell lung carcinoma the observation of a group of *bcl*-2-positive tumours with relatively slow disease progression suggests a role for *bcl*-2 expression as an initial oncogenic effect leading to less aggressive tumour growth as observed in follicular lymphoma (Pezzella *et al.*, 1993). The observed difference in independent prognostic power for *bcl*-2 expression between axillary node-negative and axillary node-positive patients in our series is extremely interesting and needs further elucidation. *In vitro* and *in vivo* studies have demonstrated a role for the bcl-2 protein in the prevention of apoptosis induced by anti-cancer drugs (Campos *et al.*, 1993; Miyashita and Reed, 1993). *Bcl*-2 transfection has been demonstrated to confer resistance to anti-cancer agents by non-conventional drug-resistance pathways (Fisher *et al.*, 1993). A recent study in breast cancer has reported bcl-2 immunostaining to be a better predictor for response to systemic endocrine therapy than oestrogen receptor status in a limited series of patients who only received endocrine therapy following primary surgery (Gee *et al.*, 1994). In contrast to our findings, Joensuu *et al.* (1994) could not demonstrate a prognostic role for *bcl*-2 expression on overall survival in axillary node-positive breast cancer patients primarily treated by radical surgery with or without locoregional radiotherapy and in whom no adjuvant therapy was administered. These data and our study results strongly suggest a role for *bcl*-2 expression as a predictor for response to chemotherapy or endocrine therapy in breast cancer patients. However, investigating such interactions in our study population is problematic because of the need for subgroup analysis in limited numbers of patients and because of the heterogeneous character of second-line and third-line treatment in those patients developing locoregional disease relapse and/or distant metastases which consists of various combinations of chemotherapy and/or endocrine therapy and/or radiotherapy. Larger patient groups with better standardised curative and palliative treatment protocols are needed to study the role of *bcl*-2 as a modulator of response to chemotherapy or endocrine therapy in breast cancer.

To what extent *bcl*-2 expression influences apoptotic cell death rate in solid neoplasms and its final effect on disease progression remains to be resolved. Recently, cDNAs have been cloned for several novel human genes, revealing a family of bcl-2 related proteins. The bax protein has been demonstrated to promote apoptosis by opposing bcl-2's function through heterodimerisation (Oltvai *et al.*, 1993). Bcl-$x_L$ has been shown to inhibit apoptosis, whereas its shorter alternative splice form bcl-$x_S$, promotes apoptosis (Boise *et al.*, 1993). The *MCL*1 gene and the *A*1 gene have been demonstrated to have sequence similarity with the *bcl*-2 gene, although their function remains unknown (Kozopas *et al.*, 1993; Lin *et al.*, 1993). Other non-bcl-2-related proteins playing a role in the regulation of programmed cell death have been isolated (Gagliardini *et al.*, 1993; Nakashima *et al.*, 1993).

Other important oncogenes and tumour-suppressor genes play a role in the regulation of apoptosis. c-*myc*, although generally considered as an important element in proliferation control, induces apoptosis in specific conditions (Evan *et al.*, 1992; Shi *et al.*, 1992). The action of c-*myc* as a stimulator of both mitogenesis and apoptosis appears to be dependent on the presence of *bcl*-2 (Bissonnette *et al.*, 1992; Fanidi *et al.*, 1992). Wild-type p53 has been demonstrated to be able to induce apoptosis (Yonis-Rouach *et al.*, 1991). The *bcl*-2 gene is a transcriptional target for wild-type p53. Wild-type p53 decreases bcl-2 protein levels and increases bax protein levels *in vitro* and *in vivo*, explaining the ability of p53 to induce apoptotic cell death (Miyashita *et al.*, 1994*a,b*). Furthermore, in breast cancer cell lines mutant p53 induces down-regulation of *bcl*-2 at both mRNA and protein level (Haldar *et al.*, 1994). The critical role played by p53 in the carcinogenesis of human breast cancer is well documented. About 30–50% of human breast cancers carry a mutant p53 gene and about an additional 30% carry non-functional wild-type p53 sequestered in the cytoplasm of tumour cells (Moll *et al.*, 1992). The presence of mutated or overexpressed p53 has been associated with poor prognostic markers, such as high histopathological grading, high levels of Ki67 or EGFR and the absence of hormone receptors, as well as with shortened disease-free survival and/or overall survival (Cattoretti *et al.*, 1988; Isola *et al.*, 1992; Poller *et al.*, 1992; Thor *et al.*, 1992; Allred *et al.*, 1993). The inverse correlation between *bcl*-2 expression and the presence of immunohistochemically demonstrable mutant p53 observed in breast cancer could reflect down-regulation of *bcl*-2 by mutant p53 (Doglioni *et al.*, 1994; Joensuu *et al.*, 1994; Leek *et al.*, 1994; Silvestrini *et al.*, 1994).

We conclude absence of *bcl*-2 expression to be an independent marker of poor prognosis in axillary node-positive breast cancer. Absence of *bcl*-2 expression in invasive ductal breast carcinoma possibly reflects down-regulation of the *bcl*-2 gene by mutant p53. Our results suggest a potentially important role for *bcl*-2 as a modulator of response to adjuvant therapy in breast cancer.

**Acknowledgements**

This work was supported by grants from the Belgian Cancer Association (Vereniging voor Kankerbestrijding and Door en Voor Kanker.

### References

AKAO Y, OTSUKI Y, KATAOKA S, ITO Y AND TSUJIMOTO Y. (1994). Multiple subcellular localization of bcl-2: detection in nuclear outer membrane, endoplasmic reticulum membrane, and mitochondrial membranes. *Cancer Res.*, **54**, 2468–2471.

ALLRED DC, CLARK GM, ELLEDGE R, FUQUA SAW, BROWN RW, CHAMNESS GC, OSBORNE CK AND McGUIRE WL. (1993). Association of p53 protein expression with tumor cell proliferation rate and clinical outcome in node-negative breast cancer. *J. Natl Cancer Inst.*, **85**, 200–206.

ALLSOPP TE, WYATT S, PATERSON HF AND DAVIES AM. (1993). The proto-oncogene *bcl*-2 can selectively rescue neurotrophic factor-dependent neurons from apoptosis. *Cell*, **73**, 295–307.

BHARGAVA V, KELL DL, VAN DE RIJN M AND WARNKE RA. (1994). Bcl-2 immunoreactivity in breast carcinoma correlates with hormone receptor positivity. *Am. J. Pathol.*, **145**, 535–540.

BISSONNETTE RP, ECHEVERRI F, MAHBOUBI A AND GREEN DR. (1992). Apoptotic cell death induced by c-myc is inhibited by bcl-2. *Nature*, **359**, 552–554.

BLOOM HJG AND RICHARDSON WW. (1957). Histological grading and prognosis in breast cancer. *Br. J. Cancer*, **11**, 359-377.

BOISE LH, GONZALEZ-GARCIA M, POSTEMA CE, DING L, LINDSTEN T, TURKA LA, MAO X, NUNEZ G AND THOMPSON CB. (1993). Bcl-x, a bcl-2-related gene that functions as a dominant regulator of apoptotic cell death. *Cell*, **74**, 597-608.

CAMPOS L, ROUAULT J-P, SABIDO O, ORIOL P, ROUBI N, VASSELON C, ARCHAMBAUD E, MAGAUD J-P AND GUYOTAT D. (1993). High expression of bcl-2 protein in acute myeloid leukemia cells is associated with poor response to chemotherapy. *Blood*, **81**, 3091-3096.

CATTORETTI G, RILKE F, ANDREOLA S, D'AMATO L AND DELIA D. (1988). P53 expression in breast cancer. *Int. J. Cancer*, **41**, 178-183.

CATTORETTI G, BECKER MHG, KEY G, DUCHROW M, SCHLUTER C, GALLE J AND GERDES J. (1992). Monoclonal antibodies against recombinant parts of the Ki-67 antigen (MIB 1 and MIB 3) detect proliferating cells in microwave-processed formalin-fixed paraffin sections. *J. Pathol.*, **168**, 357-363.

CHAN WK, POULSOM R, LU QL, PATEL K, GREGORY W, FISHER CJ AND HANBY AM. (1993). Bcl-2 expression in invasive mammary carcinoma: correlation with apoptosis, hormone receptors and p53 expression. *J. Pathol.*, **169**, 153$^{A}$.

CHEN-LEVY Z AND CLEARY ML. (1990). Membrane topology of the bcl-2 proto-oncogenic protein demonstrated *in vitro*. *J. Biol. Chem.*, **265**, 4929-4933.

CLEARY ML, SMITH SD AND SKLAR J. (1986). Cloning and structural analysis of cDNAs for bcl-2 and a hybrid bcl-2/immunoglobulin transcript resulting from the t(14;18) translocation. *Cell*, **47**, 19-28.

COX DR. (1972). Regression models and life tables. *J.R. Stat. Soc.*, **34**, 187-220.

DE JONG D, PRINS FA, MASON DY, REED JC, VAN OMMEN GB AND KLUIN PM. (1994). Subcellular localization of the bcl-2 protein in malignant and normal lymphoid cells. *Cancer Res.*, **54**, 256-260.

DOGLIONI C, DEI TOS AP, LAURINO L, CHIARELLI C, BARBARESCHI M AND VIALE G. (1994). The prevalance of bcl-2 immunoreactivity in breast carcinomas and its clinicopathological correlates, with particular reference to oestrogen receptor status. *Virchows Archiv. A, Pathol. Anat.*, **424**, 47-51.

EVAN GI, WYLLIE AH, GILBERT CS, LITTLEWOOD TD, LAND H, BROOKS M, WATERS CM, PENN LZ AND HANCOCK DC. (1992). Induction of apoptosis in fibroblasts by c-myc protein. *Cell*, **69**, 119-128.

FANIDI A, HARRINGTON EA AND EVAN GI. (1992). Cooperative interaction between *c-myc* and *bcl-2* proto-oncogenes. *Nature*, **359**, 554-556.

FISHER TC, MILNER AE, GREGORY CD, JACKMAN AL, AHERNE GW, HARTLEY JA, DIVE C AND HICKMAN JA. (1993). Bcl-2 modulation of apoptosis induced by anticancer drugs: resistance to thymidylate stress is independent of classical resistance pathways. *Cancer Res.*, **53**, 3321-3326.

GAGLIARDINI V, FERNANDEZ P-A, LEE RKK, DREXLER HCA, ROTELLO RJ, FISHMAN MC AND YUAN J. (1994). Prevention of vertebrate neuronal death by the crmA gene. *Science*, **263**, 826-828.

GARCIA I, MARTINOU I, TSUJIMOTO Y AND MARTINOU J-C. (1992). Prevention of programmed cell death of sympathetic neurons by the bcl-2 proto-oncogene. *Science*, **258**, 302-304.

GEE JMW, ROBERTSON JFR, ELLIS OA, WILLSHER P, McCLELLAND RA, HOYLE HB, KYME SR, FINLAY P, BLAMEY RW AND NICHOLSON RI. (1994). Immunocytochemical localization of bcl-2 protein in human breast cancers and its relationship to a series of prognostic markers and response to endocrine therapy. *Int. J. Cancer*, **59**, 619-628.

HALDAR S, NEGRINI M, MONNE M, SABBIONI S AND CROCE CM. (1994). Down-regulation of bcl-2 by p53 in breast cancer cells. *Cancer Res.*, **54**, 2095-2097.

HOCKENBERY D, NUNEZ G, MILLIMAN C, SCHREIBER RD AND KORSMEYER SJ. (1990). Bcl-2 is an inner mitochondrial membrane protein that blocks programmed cell death. *Nature*, **348**, 334-336.

HOCKENBERY DM, ZUTTER M, HICKEY W, NAHM M AND KORSMEYER SJ. (1991). Bcl-2 protein is topographically restricted in tissues characterized by apoptotic cell death. *Proc. Natl Acad. Sci. USA*, **88**, 6961-6965.

ISOLA J, VISAKORPI T, HOLLI K AND KALLIONIEMI O-P. (1992). Association of overexpression of tumor suppressor protein p53 with rapid cell proliferation and poor prognosis in node-negative breast cancer patients. *J. Natl Cancer Inst.*, **84**, 1109-1114.

INTERNATIONAL UNION AGAINST CANCER. (1992). *TNM Atlas. Illustrated Guide to the TNM/pTNM Classification of Malignant Tumours*, 4th edn, 2nd revision. Springer: Berlin.

JOENSUU H, PYLKKANEN L AND TOIKKANEN S. (1994). Bcl-2 protein expression and long-term survival in breast cancer. *Am. J. Pathol.*, **145**, 1191-1198.

KAPLAN EL AND MEIER P. (1958). Non-parametric estimation from incomplete observations. *J. Am. Stat. Assoc.*, **53**, 457-481.

KORSMEYER SJ. (1992). Bcl-2 initiates a new category of oncogenes: regulators of cell death. *Blood*, **80**, 879-886.

KOZOPAS KM, YANG T, BUCHAN HL, ZHOU P AND CRAIG RW. (1993). MCL1, a gene expressed in programmed myeloid cell proliferation, has sequence similarity to bcl-2. *Proc. Natl Acad. Sci. USA*, **90**, 3516-3520.

KRAJEWSKI S, TANAKA S, TAKAYAMA S, SCHIBLER MJ, FENTON W AND REED JC. (1993). Investigation of the subcellular distribution of the bcl-2 oncoprotein: residence in the nuclear envelope, endoplasmatic reticulum, and outer mitochondrial membranes. *Cancer Res.*, **53**, 4701-4714.

LEBRUN DP, WARNKE RA AND CLEARY ML. (1993). Expression of bcl-2 in fetal tissues suggests a role in morphogenesis. *Am. J. Pathol.*, **142**, 743-753.

LEEK RD, KAKLAMANIS L, PEZZELLA F, GATTER KC AND HARRIS AL. (1994). Bcl-2 in normal human breast and carcinoma, association with oestrogen receptor-positive, epidermal growth factor receptor-negative tumours and *in situ* cancer. *Br. J. Cancer*, **69**, 135-139.

LIN EY, ORLOFSKY A, BERGER MS AND PRYSTOWSKY MB. (1993). Characterization of A1, a novel hemopoietic-specific early-response gene with sequence similarity to bcl-2. *J. Immunol.*, **151**, 1979-1988.

LU Q-L, POULSOM R, WONG L AND HANBY AM. (1993). Bcl-2 expression in adult and embryonic non-haematopoietic tissues. *J. Pathol.*, **169**, 431-437.

McDONNELL TJ, DEANE N, PLATT FM, NUNEZ G, JAEGER U, McKEARN JP AND KORSMEYER SJ. (1989). Bcl-2-immunoglobulin transgenic mice demonstrate extended B-cell survival and follicular lymphoproliferation. *Cell*, **57**, 79-88.

MIYASHITA T AND REED JC. (1993). Bcl-2 oncoprotein blocks chemotherapy-induced apoptosis in a human leukemia cell line. *Blood*, **81**, 151-157.

MIYASHITA T, HARIGAI M, HANADA M AND REED JC. (1994*a*). Identification of a p53-dependent negative response element in the bcl-2 gene. *Cancer Res.*, **54**, 3131-3135.

MIYASHITA T, KRAJEWSKI S, KRAJEWSKA M, WANG HG, LIN HK, LIEBERMANN DA, HOFFMAN B AND REED JC. (1994*b*). Tumor suppressor p53 is a regulator of *bcl-2* and *bax* gene expression in vitro and in vivo. *Oncogene*, **9**, 1799-1805.

MOLL UM, RIOU G AND LEVINE AJ. (1992). Two distinct mechanisms alter p53 in breast cancer: mutation and nuclear exclusion. *Proc. Natl Acad. Sci. USA*, **89**, 7262-7266.

NAKASHIMA T, SEKIGUCHI T, KURAOKA A, FUKUSHIMA K, SHIBATA Y, KOMIYAMA S AND NISHIMOTO T. (1993). Molecular cloning of a human cDNA encoding a novel protein, DAD1, whose defect causes apoptotic cell death in hamster BHK21 cells. *Mol. Cell. Biol.*, **13**, 6367-6374.

NATHAN B, GUSTERSON B, JADAYEL D, O'HARE M, ANBAZHAGAN R, JAYATILAKE H, EBBS S, MICKLEM K, PRICE K, GELBER R, REED R, SENN H-J, GOLDHIRSCH A AND DYER MJS. (1994). Expression of BCL-2 in primary breast cancer and its correlation with tumour phenotype. *Ann. Oncol.*, **5**, 409-414.

NUNEZ G, LONDON L, HOCKENBERRY D, ALEXANDER M, McKEARN JP AND KORSMEYER SJ. (1990). Deregulated *bcl-2* gene expression selectively prolongs survival of growth factor-deprived hemopoietic cell lines. *J. Immunol.*, **144**, 3602-3610.

OLTVAI ZN, MILLIMAN CL AND KORSMEYER SJ. (1993). *Bcl-2* heterodimerizes in vivo with a conserved homolog, bax, that accelerates programmed cell death. *Cell*, **74**, 609-619.

PEZZELLA F, TSE AGD, CORDELL JL, PULFORD KAF, GATTER KC AND MASON DY. (1990). Expression of the bcl-2 oncogene protein is not specific for the 14; 18 translocation. *Am. J. Pathol.*, **137**, 225-232.

PEZZELLA F, JONES M, RALFKIAER E, ERSBOLL J, GATTER KC AND MASON DY. (1992). Evaluation of bcl-2 protein expression and 14; 18 translocation as prognostic factors in follicular lymphoma. *Br. J. Cancer*, **65**, 87-89.

PEZZELLA F, TURLEY H, KUZU I, TUNGEKAR MF, DUNNILL MS, PIERCE CB, HARRIS A, GATTER KC AND MASON DY. (1993). Bcl-2 protein in non-small-cell lung carcinoma. *N. Engl. J. Med.*, **329**, 690-694.

POLLER DN, HUTCHINGS CE, GALEA M, BELL JA, NICHOLSON RA, ELSTON CW, BLAMEY RW AND ELLIS IO. (1992). P53 protein expression in human breast carcinoma: relationship to expression of epidermal growth factor receptor, c-erbB-2 protein overexpression, and oestrogen receptor. *Br. J. Cancer*, **66**, 583–588.

REED JC, STEIN C, SUBASINGHE C, HALDAR S, CROCE CM, YUM S AND COHEN J. (1990). Antisense-mediated inhibition of *bcl-2* protooncogene expression and leukemic cell growth and survival: comparisons of phosphodiester and phosphorothioate oligodeoxynucleotides. *Cancer Res.*, **50**, 6565–6570.

SHI Y, GLYNN JM, GUILBERT LJ, COTTER TG, BISSONNETTE RP AND GREEN DR. (1992). Role for c-myc in activation-induced apoptotic cell death in T cell hybridomas. *Science*, **257**, 212–214.

SILVESTRINI R, VENERONI S, DAIDONE MG, BENINI E, BORACCHI P, MEZZETTI M, DI FRONZO G, RILKE F AND VERONESI U. (1994). The bcl-2 protein: a prognostic indicator strongly related to p53 protein in lymph-node-negative breast cancer patients. *J. Natl Cancer Inst.*, **86**, 499–504.

SIMON R AND ALTMAN RG. (1994). Statistical aspects of prognostic factor studies in oncology. *Br. J. Cancer*, **69**, 979–985.

STRASSER A, HARRIS AL AND CORY S. (1991). Bcl-2 transgene inhibits T cell death and perturbs thymic self-censorship. *Cell*, **67**, 889–899.

THOR AD, MOORE II DH, EDGERTON SM, KAWASAKI ES, REIHSAUS E, LYNCH HT, MARCUS JN, SCHWARTZ L, CHEN L-C, MAYALL BH AND SMITH HS. (1992). Accumulation of p53 tumor suppressor gene protein: an independent marker of prognosis in breast cancers. *J. Natl. Cancer Inst.*, **84**, 845–855.

TUSJIMOTO Y AND CROCE CM. (1986). Analysis of the structure, transcripts, and protein products of *bcl-2*, the gene involved in human follicular lymphoma. *Proc. Natl Acad. Sci. USA*, **83**, 5214–5218.

TUSJIMOTO Y, FINGER LR, YUNIS J, NOWELL PC AND CROCE CM. (1984). Cloning of the chromosome breakpoint of neoplastic B cells with the t(14; 18) chromosome translocatioin. *Science*, **226**, 1097–1099.

VAUX DL, CORY S AND ADAMS JM. (1988). Bcl-2 gene promotes haemopoietic cell survival and cooperates with c-myc to immortalize pre-B cells. *Nature*, **335**, 440–442.

YONISH-ROUACH E, RESNITZKY D, LOTEM J, SACHS L, KIMCHI A AND OREN M. (1991). Wild-type p53 induces apoptosis of myeloid leukaemic cells that is inhibited by interleukin-6. *Nature*, **352**, 345–347.